# Prospective evaluation of non-invasive saliva specimens for the diagnosis of syphilis and molecular surveillance of *Treponema pallidum*

**DOI:** 10.1128/jcm.00809-24

**Published:** 2024-11-06

**Authors:** Kazuo Imai, Akihiro Sato, Masashi Tanaka, Yuki Ohama, Shu-ichi Nakayama, Ryuha Omachi, Keita Takeuchi, Norihito Tarumoto, Mieko Tokano, Shigefumi Mesaki, Takuya Maeda, Yukihiro Akeda

**Affiliations:** 1 Department of Clinical Laboratory, Saitama Medical University Hospital, Saitama, Japan; 2 Department of Infectious Disease and Infection Control, Saitama Medical University, Saitama, Japan; 3 KARADA Internal Medicine Clinic, Tokyo, Japan; 4 KARADA Internal Medicine Clinic Shibuya, Tokyo, Japan; 5 Department of Bacteriology I, National Institute of Infectious Diseases, Tokyo, Japan; Maine Medical Center Department of Medicine, Portland, Maine, USA

**Keywords:** syphilis, *Treponema*, saliva, PCR, LAMP, MLST

## Abstract

The promising diagnostic performance of molecular testing for syphilis using saliva and urine samples has been reported; however, further evaluation of its possible application for diagnosis and molecular surveillance is required. In addition, the development of a rapid and easy-to-perform molecular test for syphilis is important for its use in the clinical setting. We comprehensively evaluated the diagnostic and surveillance performance of two novel loop-mediated isothermal amplification (LAMP) assays using saliva and urine samples. Saliva, urine, and whole blood were collected from patients who underwent serological testing for syphilis at outpatient clinics. *Treponema pallidum* DNA in specimens was detected using quantitative PCR (qPCR), nested PCR, and novel LAMP assays. *T. pallidum* genotyping was conducted by multi-locus sequence typing (MLST). Of the 163 patients recruited, 98 were diagnosed with syphilis (primary: *n* = 35; secondary: *n* = 40; latent: *n* = 23). qPCR showed the highest sensitivity among the molecular tests performed with a sensitivity of 54.1% and 30.3% for all syphilis patients using saliva and urine samples, respectively. A novel method of LAMP combined with dry reagents and crude DNA extraction (Dry-LAMP) showed a probit detection limit of 37.4 copies/reaction within 45 min. The agreement rate between Dry-LAMP and qPCR for saliva was 95.7% (*κ* coefficient 0.90). The *T. pallidum* genotype was identified in 48 patients by MLST using saliva samples. Molecular analysis of saliva could be used as a supplementary diagnostic test for syphilis and molecular surveillance of the *T. pallidum* genotype. Dry-LAMP is expected to be helpful in the clinical diagnosis of syphilis.

## INTRODUCTION

Syphilis is a sexually transmitted disease caused by the bacterium *Treponema pallidum* subsp. *pallidum*. Syphilis has a wide variety of clinical presentations, including genital ulcers, skin lesions, hepatitis, meningitis, aortic disease, and neurologic syndromes. Syphilis in pregnant women can lead to fetal loss, stillbirth, neonatal death, and congenital infections in neonates. The number of patients with syphilis has increased significantly worldwide in the past few decades (1). To prevent the transmission of syphilis, it is necessary to identify infected individuals through prompt diagnostic testing, provide appropriate treatment for positive cases, and offer comprehensive sexual health education (2). In addition, molecular surveillance of *T. pallidum* clonality has the potential to enhance clinical care, prevention, and control efforts by contributing to a better understanding of *T. pallidum* acquisition and transmission (3). However, the efficient diagnosis and molecular surveillance of syphilis remain challenging for a number of reasons. First, most patients are asymptomatic and are identified only during screening. Second, the diagnosis of syphilis relies on serologic testing (treponemal and nontreponemal tests), and patients are often missed within the period between the initial *T. pallidum* infection and the appearance of antibodies in early syphilis for serologic testing or because they refuse to provide blood samples. Third, it is difficult to culture *T. pallidum in vitro*. Fourth, lesions of primary and secondary syphilis contain low loads of *T. pallidum* (4).

Recently, several reports have demonstrated that *T. pallidum* DNA can be detected in non-invasive specimens, such as oral fluid (5, 6), saliva (7), urine (8), and semen (9). Wang et al. (7, 8) demonstrated the promising diagnostic performance of self-collected saliva and urine specimens by using nested PCR (nPCR) and droplet digital PCR. However, it is necessary to further evaluate the performance of molecular testing with saliva and urine samples in the context of prospective studies in a population in which syphilis is suspected in the clinical setting. In addition, an alternative molecular test that is rapid, easy to perform, and cost-effective is desirable for the clinical diagnosis of syphilis instead of nPCR or droplet digital PCR, which require time-consuming protocols, special equipment, and highly skilled laboratory technicians. Loop-mediated isothermal amplification (LAMP) (10), which does not require expensive devices or instruments, can be highly convenient to use as a point-of-care test in the clinical setting, especially in resource-limited areas (11, 12). Several LAMP assays for syphilis have been reported, but their diagnostic performance with non-invasive specimens has not been evaluated (13–18). In addition, the reported assays have major limitations in terms of their clinical usage, including low sensitivity, long sample preparation and reaction times, and requirement for special equipment and a cold chain for the storage of reagents (13–18). For molecular surveillance, strain typing methods by Enhanced Centers for Disease Control and Prevention typing (19), multi-locus sequence typing (MLST) (20), and whole-genome sequencing (21) are widely applied in patients such as those with genital ulcers. However, there is still a gap in our knowledge regarding the usefulness of non-invasive specimens from patients with limited clinical information (20–22).

Therefore, we comprehensively evaluated the diagnostic and surveillance performance of molecular tests, including two novel LAMP assays and MLST, using non-invasive saliva and urine specimens.

## MATERIALS AND METHODS

### Study design, patients, and sample collection

**Fig 1:**
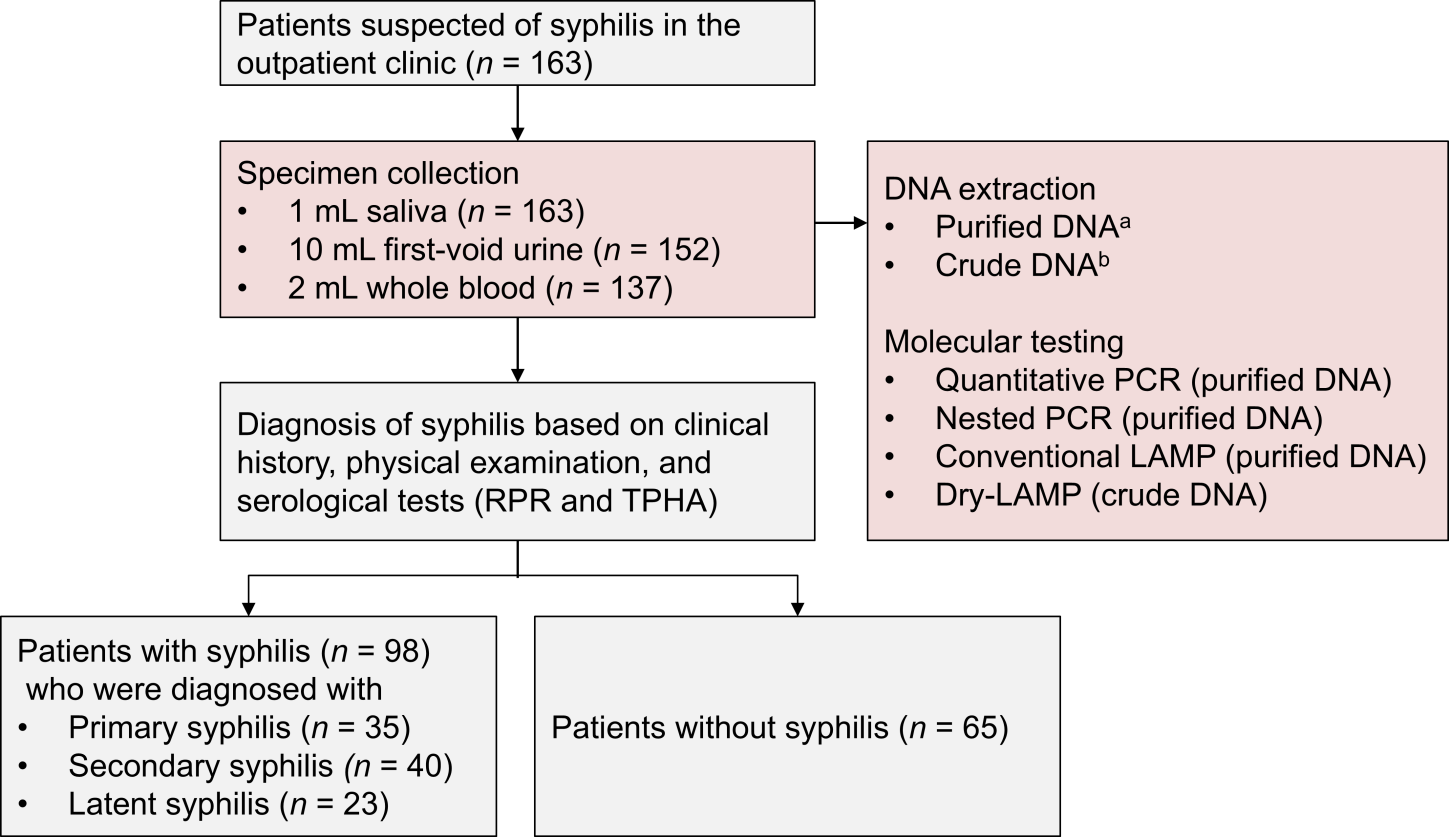
Study patients, sampling, and testing. Superscript “a” indicates DNA was extracted using a QIAamp DNA Mini Kit (Qiagen) for saliva and whole blood and a QIAamp Viral RNA Mini Kit (Qiagen) for urine. Superscript “b” indicates DNA was extracted using the MightyPrep Reagent for DNA (Takara Bio, Inc.). RPR, rapid plasma regain; TPHA, *Treponema pallidum* hemagglutination assay.

We conducted a multicenter prospective study at Saitama Medical University Hospital (Moroyama, Japan) and at the KARADA Internal Medicine Clinic and KARADA Internal Medicine Clinic Shibuya (Tokyo, Japan) between 21 May 2023 and 30 April 2024. Adult patients (≥18 years old) who were suspected of syphilis in the outpatient setting were enrolled in this study according to the following criteria: (i) persons with clinical symptoms indicative of syphilis (genital nodules or redness, ulcers, inguinal lymphadenopathy, and skin and mucocutaneous lesions), and (ii) persons who have had sexual contact with a person infected with syphilis or with multiple or new sex partners. At the time of serological testing for syphilis, 1 mL of self-collected saliva, 10 mL of first-void urine, and 2 mL of whole blood were collected from the patients (Fig. 1). The saliva specimens were collected without restriction on timing or food intake. The specimens were stored at −80°C until DNA extraction. Patient information was collected retrospectively from the hospital’s electronic medical records.

### Definitions

The clinical stages of syphilis were classified according to the following standard criteria: (i) primary syphilis: clinical manifestation of chancres and/or ulcers with a positive serum rapid plasma regain (RPR) test (>1 IU/mL) and *T. pallidum* hemagglutination assay (TPHA) (>6 IU/mL) during a 2-month follow-up period; (ii) secondary syphilis: skin or mucocutaneous lesions with a positive serum RPR test and TPHA; (iii) latent syphilis: asymptomatic with a positive serum RPR test and TPHA; and (iv) for patients with a history of treatment for syphilis, those with seroconversion of RPR or a new fourfold elevation in the titer of the RPR test were considered as newly infected with syphilis. Patients who did not meet any of these criteria were diagnosed with non-syphilis diseases.

### Sample preparation and DNA extraction

Purified DNA was extracted from 200 µL saliva and whole blood samples using a QIAamp DNA Mini Kit (Qiagen, Hilden, Germany), and the DNA was eluted in 100 µL of 10 mM Tris-HCl buffer, pH 8.5. First-void urine samples (10 mL) were centrifuged at 3,000 × *g* for 15 min at room temperature, and the sediment was resuspended in 1 mL supernatant. DNA was extracted from 140 µL of the suspension using a QIAamp Viral RNA Mini Kit (Qiagen), and the DNA was eluted in 70 µL of 10 mM Tris-HCl buffer, pH 8.5. Residual specimens and DNA were stored at −80°C until testing. Crude DNA was extracted using the MightyPrep Reagent for DNA (Takara Bio, Inc., Shiga, Japan). The saliva samples (50 µL) were centrifuged at 15,000 × *g* for 3 min at room temperature, and the sediment was resuspended in 50 µL lysis buffer and then heated at 95°C for 10 min. The concentrated urine and blood samples (50 µL) were mixed in 50 µL lysis buffer and then heated at 95°C for 10 min. The suspension was centrifuged at 15,000 × *g* for 3 min at room temperature, and the supernatant was used in testing within 30 min.

### qPCR of *tp47* and *polA*

qPCR amplification of the *tp47* (*tp0574*) (23) and *polA* (*tp0105*) (24) genes of *T. pallidum* was performed using the TaqMan Fast Advanced Master Mix for qPCR (Applied Biosystems, Waltham, MA, USA). The primer sequences are shown in Table S1. The final reaction volume was 20 µL, comprising 2 µL of purified DNA sample. Thermal cycling consisted of 50°C for 2 min, 95°C for 30 s, and 45 cycles of denaturation at 95°C for 1 s and annealing/extension at 60°C for 20 s. A positive result, which was determined according to a Ct value of <45, with either or both of the primer and probe sets indicated the presence of *T. pallidum* DNA. Tenfold serial dilutions of AMPLIRUN Treponema DNA Control (Vircell S.L., Granada, Spain) ranging from 2.0 to 2,000 copies/reaction were used to estimate the *T. pallidum* DNA copy number in the specimens based on the standard curve method. The reactions were conducted in duplicate.

### nPCR of *tp47* and *polA*

nPCR amplification of the *tp47* (25) and *polA* genes (26) of *T. pallidum* was performed using KOD One PCR Master Mix (Toyobo Co., Ltd., Osaka, Japan). The primer sequences are shown in Table S1. The final reaction volume was 20 µL, comprising 2 µL of purified DNA and 100-fold diluted first PCR product for the first and second PCR, respectively. Thermal cycling was conducted with the following conditions: *polA*: first PCR, 98°C for 30 s and 40 cycles at 98°C for 10 s and 68°C for 5 s; second PCR, 98°C for 30 s and 40 cycles at 98°C for 10 s, 62°C for 5 s, and 68°C for 1 s. *tp47*: first PCR, 98°C for 30 s and 40 cycles at 98°C for 10 s, 58°C for 5 s, and 68°C for 1 s; second PCR, 98°C for 30 s and 40 cycles at 98°C for 10 s, 60°C for 5 s, and 68°C for 1 s. PCR products were analyzed by electrophoresis in a 2% agarose gel with ethidium bromide under an ultraviolet transilluminator. A positive result with either or both of the primer sets indicated the presence of *T. pallidum* DNA. The reactions were conducted in duplicate.

### Development of the conventional LAMP method

The oligonucleotide LAMP primers for the detection of the *T. pallidum* basic membrane protein (*bmp*) gene were designed using online LAMP primer design software (PrimerExplorer 5, http://primerexplorer.jp/index.html; Eiken Chemical Co., Ltd., Tokyo, Japan). The primer sequences are shown in Table S1. The reactions were performed using a LAMP MASTER for Turbidity (Nippon Gene Co., Ltd., Tokyo, Japan). The final reaction volume was 25 µL, comprising 40 pmol FIP primer and 40 pmol BIP primer, 20 pmol each loop primer, 5 pmol F3 and B3 primers, 12.5 µL of 2× reaction mix, and 10 µL DNA sample. The LAMP amplicons were detected by real-time measurements of turbidity using a Loopamp real-time turbidimeter (LA-200; Eiken Chemical Co., Ltd.). The reactions were carried out at 67°C for 45 min. Tenfold serial dilutions of AMPLIRUN *Treponema* DNA Control (Vircell S.L.) ranging from 2.0 to 2,000 copies/reaction were produced to evaluate the specificity of the assay. Melting curve analysis was conducted after the LAMP reactions using a LAMP MASTER for Fluorescence with a fluorescent dye (Nippon Gene Co., Ltd.) instead of a LAMP MASTER for Turbidity in triplicate. Additionally, the cross-reactivity of LAMP to DNA from 56 bacteria, fungi, and viral pathogens was examined in duplicate (Table S2).

### Development of the Dry-LAMP assay

LAMP reactions were performed by using DryADD LAMP Master Mix (Nippon Gene Co., Ltd.). The LAMP primers designed for the conventional LAMP assay were used for the Dry-LAMP assay (Table S1). The final reaction volume was 25 µL, comprising 40 pmol FIP primer and 40 pmol BIP primer, 20 pmol each loop primer, 5 pmol F3 and B3 primers, 5 µL LTV Dissolve Solution, and 2 µL crude DNA sample. The LAMP amplicons were detected by real-time measurements of turbidity using a Loopamp real-time turbidimeter (LA-200) and visual observations of color changes with the naked eye under ultraviolet light.

### Determination of the probit limit of detection

Tenfold serial dilutions of AMPLIRUN *Treponema* DNA Control ranging from 2.0 to 2,000 copies/reaction were produced to determine the preliminary limit of detection for the LAMP reactions in triplicate. Subsequently, twofold serial dilutions of AMPLIRUN *Treponema* DNA Control ranging from 80 to 10 copies/reaction were produced to determine the probit limit of detection (*T. pallidum* DNA copy number at a 95% detection rate) for the LAMP reactions in 20 replicates.

### MLST and multi-locus sequence analysis

MLST (20) and multi-locus sequence analysis (MLSA) (27) were performed as described previously. The primer sequences are shown in Table S1. nPCR amplification of the *tp0136*, *tp0548*, and *tp0705* loci for MLST and *tp0548* and *tp0856* loci for MLSA was performed using KOD One PCR Master Mix (Toyobo). The amplicons were analyzed by 1% agarose gel electrophoresis with ethidium bromide staining, and the PCR products were purified using a QIAquick Gel Extraction Kit (Qiagen). Direct Sanger sequencing was performed by Eurofins Genomics (Tokyo, Japan). The sequence types of MLST were analyzed using the PubMLST BIGSdb database of *T. pallidum* (https://pubmlst.org/organisms/treponema-pallidum). The phylogenetic tree of MLSA was constructed by using MEGA 7.0 (28). The reference sequences used in this study for MLSA are listed in Table S3.

### Statistical analysis

Continuous variables with a normal distribution are expressed as mean (±standard deviation) and with a non-normal distribution as median (interquartile range). Categorical variables are expressed as numbers (%). Sensitivity and specificity with a 95% confidence interval were calculated to assess diagnostic performance in R version 4.1.2 (R Foundation for Statistical Computing, Vienna, Austria) (29). Probit analysis was performed using MedCalc statistical software (MedCalc Software Ltd., Ostend, Belgium).

## RESULTS

### Baseline characteristics of the participants

During the study period, 163 patients with suspected syphilis were enrolled, and saliva (*n* = 163), urine (*n* = 152), and whole blood (*n* = 137) samples were collected. The baseline characteristics of the study participants are shown in Table 1. The median age was 33 years (interquartile range 25–44 years), and 130 (79.8%) patients were male. The most common clinical symptom was skin rash (*n* = 59, 36.2%), followed by a genital nodule or redness (*n* = 37, 22.7%) and a genital ulcer (*n* = 29, 17.8%). Ninety-eight patients were diagnosed with syphilis (primary: *n* = 35; secondary: *n* = 40; and latent: *n* = 23) based on clinical history, physical examination, and serological tests. The remaining 65 patients were diagnosed with non-syphilis diseases.

**TABLE 1:** Clinical characteristics of the study participants^*a*^

	All patients (*n* = 163)	Patients with syphilis (*n* = 98)	Patients without syphilis (*n* = 65)
Age (years)	33 [25–44]	32 [25–44]	30 [26–43]
Male sex	130 (79.8)	71 (72.4)	59 (90.8)
Symptoms			
Genital nodule or redness	37 (22.7)	15 (15.3)	22 (33.8)
Genital ulcer	29 (17.8)	23 (23.5)	6 (9.2)
Inguinal lymphadenopathy	12 (7.4)	6 (6.1)	6 (9.2)
Any type of skin rash	59 (36.2)	40 (40.8)	19 (29.2)
Asymptomatic	34 (20.9)	23 (23.5)	14 (21.5)
Diagnosis of clinical stages of syphilis			
Primary	35 (35.7)	35 (35.7)	–^*b*^
Secondary	40 (40.8)	40 (40.8)	–
Latent	23 (23.5)	23 (23.5)	–
Serological findings			
RPR (IU/mL)	–	52.0 [9.0–91.0]	–
TPHA (IU/mL)	–	621 [218–2,863]	–
Specimen			
Saliva	163 (100)	98 (100)	65 (100)
Urine	152 (93.3)	89 (90.8)	63 (96.2)
Whole blood	137 (84.0)	76 (77.6)	61 (93.8)

^*a*^ Data are shown as median [interquartile range] or number (percentage) as appropriate.

^*b*^ –, not applicable.

### Diagnostic performance of qPCR and nPCR with saliva, urine, and whole blood samples

The sensitivity and specificity of qPCR and nPCR with clinical specimens (saliva: *n* = 163; urine: *n* = 152; whole blood: *n* = 137) for the diagnosis of syphilis are shown in Table 2. The sensitivity (95% CI) of qPCR and nPCR for all syphilis cases was 54.1% (43.7%–64.2%) and 53.1% (42.7%–63.2%) for saliva, 30.3% (21.0%–41.0%) and 25.8% (17.1%–36.2%) for urine, and 40.8% (29.7%–52.7%) and 44.7% (33.3%–56.6%) for whole blood, respectively. All saliva and urine specimens collected from patients without syphilis were negative, and specificity (95% CI) was 100% (94.3%–100%) for both qPCR and nPCR. A false-positive result for a blood specimen with nPCR was observed in a patient who was suspected of primary syphilis and was repeatedly confirmed to be negative in serological testing, but not for qPCR, resulting in a specificity (95% CI) of 100% (94.1%–100%) and 98.4% (91.2%–100%) for qPCR and nPCR in patients without syphilis, respectively.

**TABLE 2:** Diagnostic performance of quantitative PCR and nested PCR with clinical specimens

	Total	True positive	True negative	False positive	False negative	Sensitivity % (95% CI)	Specificity % (95% CI)
Saliva (*n* = 163)							
Quantitative PCR							
All patients	163	53	65	0	45	54.1 (43.7–64.2)	100 (94.5–100)
Primary	35	9	–^*a*^	–	26	25.7 (12.5–43.3)	–
Secondary	40	33	–	–	7	82.5 (67.2–92.7)	–
Latent	23	11	–	–	12	47.8 (26.8–69.4)	–
Nested PCR							
All syphilis	163	52	65	0	46	53.1 (42.7–63.2)	100 (94.5–100)
Primary	35	10	–	–	25	28.6 (14.6–46.3)	–
Secondary	40	32	–	–	8	80.0 (64.4–91)	–
Latent	23	10	–	–	13	43.5 (23.2–65.5)	–
Urine (*n* = 152)							
Quantitative PCR							
All syphilis	152	27	63	0	62	30.3 (21.0–41.0)	100 (94.3–100)
Primary	33	13	–	–	20	39.4 (22.9–57.9)	–
Secondary	37	11	–	–	26	29.7 (15.9–47.0)	–
Latent	19	3	–	–	16	15.8 (3.4–39.6)	–
Nested PCR							
All syphilis	152	23	63	0	66	25.8 (17.1–36.2)	100 (94.3–100)
Primary	33	11	–	–	22	33.3 (18.0–51.8)	–
Secondary	37	10	–	–	27	27.0 (13.8–44.1)	–
Latent	19	2	–	–	17	10.5 (1.3–33.1)	–
Blood (*n* = 137)							
Quantitative PCR							
All syphilis	137	31	61	0	45	40.8 (29.7–52.7)	100 (94.1–100)
Primary	28	10	–	–	18	35.7 (18.6–55.9)	–
Secondary	33	18	–	–	15	54.5 (36.4–71.9)	–
Latent	15	3	–	–	12	20 (4.3–48.1)	–
Nested PCR							
All syphilis	137	34	60	1	42	44.7 (33.3–56.6)	98.4 (91.2–100)
Primary	28	12	–	–	16	42.9 (24.5–62.8)	–
Secondary	33	18	–	–	15	54.5 (36.4–71.9)	–
Latent	15	4	–	–	11	26.7 (7.8–55.1)	–

^*a*^ –, not applicable.

By clinical stage, saliva showed the highest sensitivity (95% CI) in secondary syphilis (82.5%, 67.2%–92.7% for qPCR, and 80.0%, 64.4%–91.0% for nPCR). Urine showed the highest sensitivity (95% CI) in primary syphilis (39.4%, 22.9%–57.9% for qPCR and 33.3%, 18.0–51.8% for nPCR), while it showed lower sensitivity (95% CI) than saliva in secondary syphilis (29.7%, 15.9%–47.0% for qPCR and 27.0%, 13.8%–44.1% for nPCR). The sensitivity (95% CI) of saliva in latent syphilis (47.8%, 26.8%–69.4% for qPCR and 43.5%, 23.2%–65.5% for nPCR) was higher than that of urine (15.8%, 3.4%–39.6% for qPCR and 10.5%, 1.3%–33.1% for nPCR) and blood (20.0%, 4.3%–48.1% for qPCR and 26.7%, 7.8%–55.1% for nPCR).

### *T. pallidum* DNA copy number

**Fig 2:**
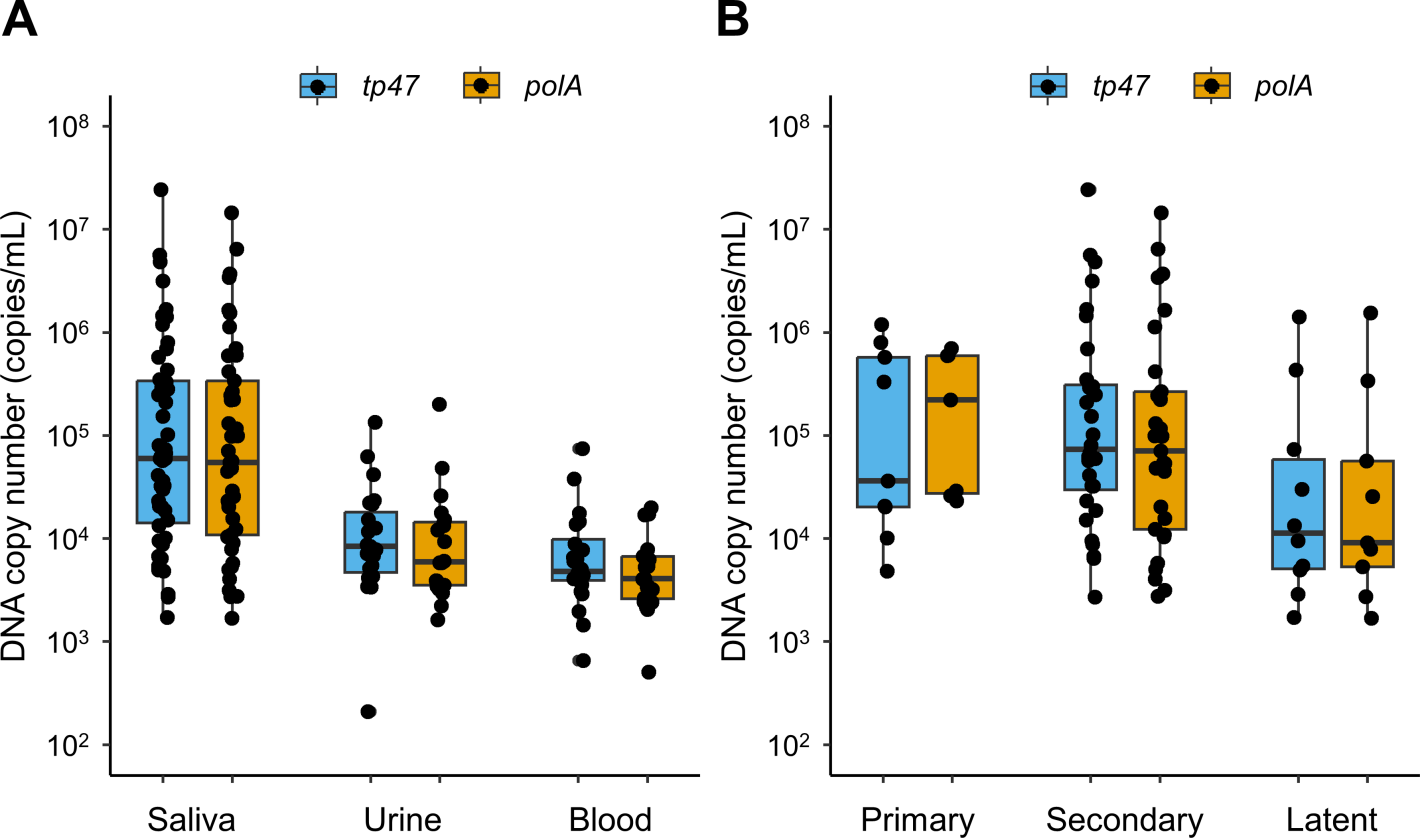
*Treponema pallidum* DNA copy number. Distribution of DNA copy number in saliva (*tp47: n* = 51; *polA: n* = 45), urine (*tp47: n* = 23; *polA: n* = 20), and blood (*tp47: n* = 24; *polA: n* = 20) (**A**) and in saliva among patients with primary (*tp47: n* = 9; *polA: n* = 7), secondary (*tp47: n* = 32; *polA: n* = 29), and latent syphilis (*tp47: n* = 10; *polA: n* = 9) (**B**). Black plots indicate the DNA copy number of each specimen.

The *T. pallidum* DNA copy number (median, interquartile range) was higher in saliva samples (59,667 copies/mL, 14,176–338,798 copies/mL for *tp47* and 54,507 copies/mL, 10,816–338,955 copies/mL for *polA*) than in urine samples (8,381 copies/mL, 4,702–18,350 copies/mL for *tp47* and 5,819, 3,447–14,216 copies/mL for *polA*) and blood samples (4,784 copies/mL, 3,930–10,052 copies/mL for *tp47* and 4,051 copies/mL, 2,595–6,670 copies/mL for *polA*) (Fig. 2A). In addition, the *T. pallidum* DNA copy number (median, interquartile range) of saliva was higher in patients with primary syphilis (36,007 copies/mL, 20,162–570,823 copies/mL for *tp47* and 220,956 copies/mL, 27,522–595,514 copies/mL for *polA*) and secondary syphilis (73,456 copies/mL, 29,959–311,672 for *tp47* and 70,506 copies/mL, 12,266–266,266 copies/mL for *polA*) than in those with latent syphilis (11,391 copies/mL, 5,077–62,479 copies/mL for *tp47* and 9,036 copies/mL, 5,320–56,422 copies/mL for *polA*) (Fig. 2B).

### Diagnostic performance of LAMP assays with saliva, urine, and whole blood samples

**Fig 3:**
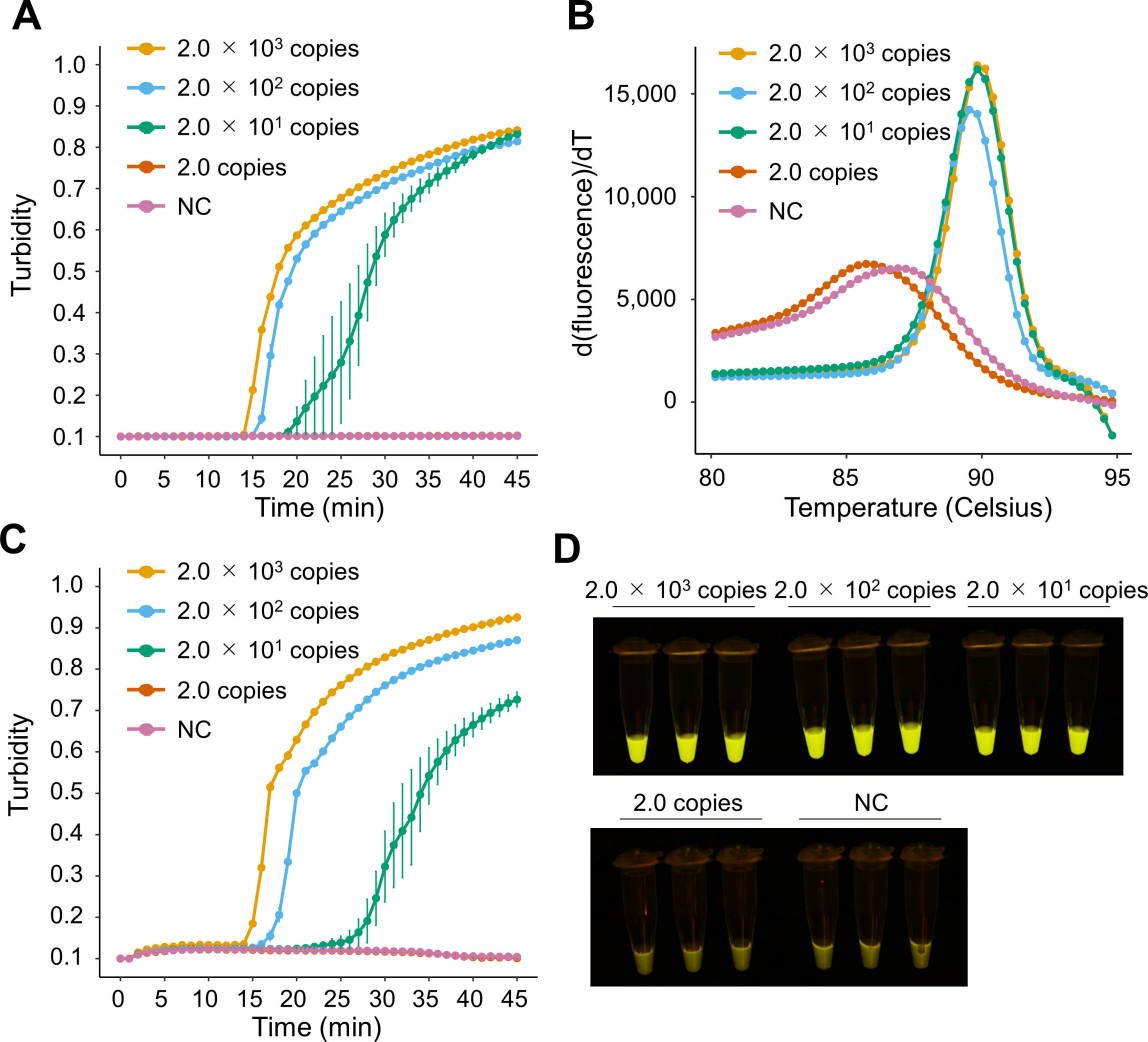
Basic performance of the loop-mediated isothermal amplification assays. The limit of detection for the conventional LAMP assay for the *bmp* gene of *Treponema pallidum* on a real-time turbidimeter (**A**), melting curve analysis of the LAMP assay with a fluorescent dye (**B**), and the preliminary limit of detection for the Dry-LAMP assay on a real-time turbidimeter (**C**) and with the naked eye under ultraviolet light (**D**). Samples of each concentration were tested in triplicate. Error bars indicate standard error intervals. NC, negative control (distilled water).

We next developed two novel LAMP assays: a conventional LAMP assay with reagents that required freeze storage with purified DNA, and a Dry-LAMP assay with dried reagent storage at room temperature with crude DNA to detect the *bmp* gene of *T. pallidum* with the aim of being used as a rapid point-of-care test. The preliminary limit of detection for the positive DNA control in the conventional LAMP assay using real-time turbidity was 20 copies/reaction at 67°C within 45 min (Fig. 3A). Melting curve analysis following the LAMP reaction with *T. pallidum* DNA produced a single peak at approximately 89.7°C (Fig. 3B). Additionally, DNA derived from another 56 pathogens, including *Treponema denticola*, was not amplified by the assay. The preliminary limit of detection for the positive DNA control did not change when using the Dry-LAMP assay with a Loopamp real-time turbidimeter (LA-200) and the naked eye (Fig. 3C and D). We additionally tested 20 replicates of serially diluted positive DNA control using the conventional and Dry-LAMP assays and performed probit analysis. The probit limit of detection was 27.5 and 37.4 copies/reaction for the conventional and Dry-LAMP assays, respectively (Table 3).

**TABLE 3:** Probit limit of detection for the LAMP assays

Assay	No. of positives/no. of replicates of *T. pallidum* DNA (copies/reaction)							Probit limit of detection (copies/reaction)
	2,000	200	80	40	20	10	2	
Conventional LAMP	3/3	3/3	20/20	20/20	16/20	9/20	0/3	27.5
Dry-LAMP	3/3	3/3	20/20	19/20	13/20	4/20	0/3	37.4

Subsequently, we assessed the performance of the LAMP assays with clinical samples. When using saliva samples, the sensitivity (95% CI) of the conventional and Dry-LAMP assays was 50% (39.7%–60.3%) and 46.9% (36.8%–57.3%) for all syphilis cases, and 28.6% (14.6%–46.3%) and 20% (8.4%–36.9%) for primary syphilis, respectively (Table 4). The sensitivity (95% CI) of both LAMP assays was 75.0% (58.8%–87.3%) in secondary syphilis and 39.1% (19.7%–61.5%) in latent syphilis. When using urine and blood samples, the sensitivity of both LAMP assays was low. The sensitivity (95% CI) of the conventional and Dry-LAMP assays for all syphilis cases was 14.6% (8.0%–23.7%) and 6.7% (2.5%–14.1%) for urine and 5.2% (1.4%–12.9%) and 1.3% (0%–7.1%) for whole blood, respectively. Specificity (95% CI) was 100% (94.5%–100%) in both LAMP assays with all clinical specimens. A high overall agreement rate was observed between the Dry-LAMP assays and the other methods in saliva, while it was low in urine and blood. The overall agreement rate between the conventional and Dry-LAMP assays was 98.2% with a *κ* coefficient of 0.96, 95.7% between Dry-LAMP and qPCR with a *κ* coefficient of 0.90, and 96.3% between Dry-LAMP and nPCR with a *κ* coefficient of 0.91 in saliva (Table S4). Disagreement between Dry-LAMP and qPCR was observed in saliva samples with a low copy number of *T. pallidum* DNA (<10^4^ copies/mL; mean 5,402 copies/mL for *tp47* and 2,955 copies/mL for *polA*). The overall agreement rate between the Dry-LAMP assays and qPCR or nPCR in urine and blood was less than 90.0%.

**TABLE 4:** Diagnostic performance of the LAMP assays with clinical specimens

	Total	True positive	True negative	False positive	False negative	Sensitivity % (95% CI)	Specificity % (95% CI)
Saliva (*n* = 163)							
Conventional LAMP assay							
All patients	163	49	65	0	49	50.0 (39.7–60.3)	100 (94.5–100)
Primary	35	10	–^*a*^	–	25	28.6 (14.6–46.3)	–
Secondary	40	30	–	–	10	75.0 (58.8–87.3)	–
Latent	23	9	–	–	14	39.1 (19.7–61.5)	–
Dry-LAMP assay							
All syphilis	163	46	65	0	52	46.9 (36.8–57.3)	100 (94.5–100)
Primary	35	7	–	–	28	20.0 (8.4–36.9)	–
Secondary	40	30	–	–	10	75.0 (58.8–87.3)	–
Latent	23	9	–	–	14	39.1 (19.7–61.5)	–
Urine (*n* = 152)							
Conventional LAMP assay							
All syphilis	152	13	63	0	76	14.6 (8.0–23.7)	100 (94.3–100)
Primary	33	7	–	–	26	21.1 (8.9–38.9)	–
Secondary	37	6	–	–	31	16.2 (6.2–32.0)	–
Latent	19	0	–	–	19	0 (0–17.6)	–
Dry-LAMP assay							
All syphilis	152	6	63	0	83	6.7 (2.5–14.1)	100 (94.3–100)
Primary	33	4	–	–	29	12.1 (3.4–28.2)	–
Secondary	37	2	–	–	35	5.4 (0.1–18.2)	–
Latent	19	0	–	–	19	0 (0–17.6)	–
Blood (*n* = 137)							
Conventional LAMP assay							
All syphilis	137	4	61	0	72	5.2 (1.4–12.9)	100 (94.1–100)
Primary	28	0	–	–	28	0 (0–12.3)	–
Secondary	33	3	–	–	30	9.1 (1.9–24.3)	–
Latent	15	1	–	–	14	6.6 (0.2–31.9)	–
Dry-LAMP assay							
All syphilis	137	1	61	0	75	1.3 (0–7.1)	100 (94.1–100)
Primary	28	0	–	–	28	0 (0–12.3)	–
Secondary	33	1	–	–	32	3.0 (0–15.8)	–
Latent	15	0	–	–	15	0 (0–21.8)	–

^*a*^ –, not applicable.

### Diagnostic performance of molecular methods using saliva combined with urine

Additionally, we calculated sensitivity when combining saliva and urine specimens excluding patients with missing data. When the combination of saliva and urine was used for diagnosis in all syphilis cases, sensitivity (95% CI) was slightly increased from 56.2% (42.3%–66.7%) to 65.2% (54.3%–75.0%) for qPCR, from 53.9% (43.0%–64.6%) to 59.6% (25.5%–60.8%) for nPCR, from 51.7% (40.8%–62.4%) to 56.2% (45.3%–66.7%) for conventional LAMP, and from 48.3% (37.6%–59.2%) to 50.6% (39.8%–61.3%) for Dry-LAMP in comparison to saliva alone (Table 5). For the clinical stage, the highest improvement of sensitivity was observed in primary syphilis. In primary syphilis, sensitivity (95% CI) was increased from 27.3% (13.3%–45.5%) to 48.5% (30.8%–66.5%) for qPCR, from 30.3% (15.6%–48.7%) to 42.4% (25.5%–60.8%) for nPCR, from 30.3% (15.6%–48.7%) to 39.4% (22.9%–57.9%) for conventional LAMP, and from 21.2% (9.0%–38.9%) to 27.3% (13.3%–45.5%) for Dry-LAMP in comparison to saliva alone (Table 5).

**TABLE 5:** Diagnostic performance of molecular methods using saliva combined with urine^*a*^

	Total	True positive	True negative	False positive	False negative	Sensitivity % (95% CI)	Specificity % (95% CI)
Quantitative PCR							
Saliva combined with urine (*n* = 152)							
All patients	152	58	63	0	31	65.2 (54.3–75.0)	100 (94.3–100)
Primary	33	16	–^*b*^	–	17	48.5 (30.8–66.5)	–
Secondary	37	32	–	–	5	86.5 (71.2–95.5)	–
Latent	19	10	–	–	9	52.6 (28.9–75.6)	–
Saliva alone (*n* = 152)							
All patients	152	50	63	0	39	56.2 (42.3–66.7)	100 (94.3–100)
Primary	33	9	–	–	24	27.3 (13.3–45.5)	–
Secondary	37	32	–	–	5	86.5 (71.2–95.5)	–
Latent	19	9	–	–	10	47.4 (24.5–71.1)	–
Nested PCR							
Saliva combined with urine (*n* = 152)							
All patients	152	53	63	0	36	59.6 (48.6–69.8)	100 (94.3–100)
Primary	33	14	–	–	19	42.4 (25.5–60.8)	–
Secondary	37	30	–	–	7	81.1 (64.8–92.0)	–
Latent	19	9	–	–	10	47.4 (24.5–71.1)	–
Saliva alone (*n* = 152)							
All patients	152	48	63	0	41	53.9 (43.0–64.6)	100 (94.3–100)
Primary	33	10	–	–	23	30.3 (15.6–48.7)	–
Secondary	37	30	–	–	7	81.1 (64.8–92.0)	–
Latent	19	8	–	–	11	42.1 (20.3–66.5)	–
Conventional LAMP assay							
Saliva combined with urine (*n* = 152)							
All patients	152	50	63	0	39	56.2 (45.3–66.7)	100 (94.3–100)
Primary	33	13	–	–	20	39.4 (22.9–57.9)	–
Secondary	37	30	–	–	7	81.1 (64.8–92.0)	–
Latent	19	7	–	–	12	36.8 (16.3–61.6)	–
Saliva alone (*n* = 152)							
All patients	152	46	63	0	43	51.7 (40.8–62.4)	100 (94.3–100)
Primary	33	10	–	–	23	30.3 (15.6–48.7)	–
Secondary	37	29	–	–	8	78.4 (61.8–90.2)	–
Latent	19	7	–	–	12	36.8 (16.3–61.6)	–
Dry-LAMP assay							
Saliva combined with urine (*n* = 152)							
All patients	152	45	63	0	44	50.6 (39.8–61.3)	100 (94.3–100)
Primary	33	9	–	–	24	27.3 (13.3–45.5)	–
Secondary	37	29	–	–	8	78.4 (61.8–90.2)	–
Latent	19	7	–	–	12	36.8 (16.3–61.6)	–
Saliva alone (*n* = 152)							
All patients	152	43	63	0	46	48.3 (37.6–59.2)	100 (94.3–100)
Primary	33	7	–	–	26	21.2 (9.0–38.9)	–
Secondary	37	29	–	–	8	78.4 (61.8–90.2)	–
Latent	19	7	–	–	12	36.8 (16.3–61.6)	–

^*a*^ Sensitivity and specificity were calculated after excluding patients with missing data.

^*b*^ –, not applicable.

### MLST and MLSA analysis of saliva

Of the 57 saliva samples that were positive for *T. pallidum* DNA by either qPCR or nPCR, 48 (primary: *n* = 8; secondary: *n* = 31; and latent: *n* = 9) were fully typed at the *tp0136*, *tp0548*, and *tp0705* loci by MLST. Among them, the 1.1.8 allelic profile (SS14-like profile) was the most common (83.3%, 40/48), followed by the 3.2.3 allelic profile (Nichols-like profile; 6.3%, 3/48) and 9.7.3 allelic profile (Nichols-like profile; 4.2%, 2/48) (Table 6). One new allelic profile (22.1.8) was discovered. In addition, we found one profile that belonged to the *T. pallidum* subsp. *endemicum* (TEN)-like group (untypable.untypable.10). MLSA using *tp0548* and *tp0856* indicated this sample was TEN (Fig. S1).

**TABLE 6:** Allelic profiles of multi-locus sequence typing among fully typed samples (*n* = 48)

No. of samples (%)	*tp0136* allele	*tp0548* allele	*tp0705* allele	Sequence type	Genetic group
40 (83.3)	1	1	8	3	SS14-like
3 (6.3)	3	2	3	6	Nichols-like
2 (4.2)	9	7	3	26	Nichols-like
1 (2.1)	37	3	1	120	SS14-like
1 (2.1)	22	1	8	124	N.A.^*b*^
1 (2.1)	Untypable	Untypable	10	Untypable	TEN^*a*^

^*a*^ According to multi-locus sequence analysis of *tp0548* and *tp0856*.

^*b*^ N.A., not applicable.

## DISCUSSION

Here, we present the usefulness of self-collected saliva samples for the diagnosis and molecular surveillance of syphilis in a prospective study. A novel Dry-LAMP assay detected *T. pallidum* DNA within 1 h with a high agreement rate with qPCR (95.7% with a *κ* coefficient of 0.90). MLST using saliva specimens could discriminate *T. pallidum* clonality among all clinical stages of syphilis.

Our study revealed that there are differences in the sensitivity of molecular tests depending on the type of specimen and the stage of syphilis. Additionally, molecular testing demonstrated high specificity across all types of specimens. Our results are in line with those reported by Wang et al. (7, 8). A small increase in sensitivity by combining saliva and urine specimens was observed, especially for primary syphilis. However, taking into account the testing cost and sample preparation time in the clinical setting, using saliva alone may be suitable for diagnosis. Another option is to select specimens according to clinical stage (saliva for secondary/latent syphilis and urine for primary syphilis). The specificity of molecular testing was high, and only one false-positive result was observed in nPCR for a blood specimen collected from a patient who was repeatedly found to be negative in serological testing. Molecular testing of non-invasive specimens may contribute to a supportive diagnosis and treatment, for example, for patients with reinfection or asymptomatic infection, which are difficult to determine by single serological tests. In addition, it will enhance prompt syphilis testing for individuals who are reluctant to provide blood samples.

In this study, we developed and evaluated a novel Dry-LAMP assay using dry reagents, which can be stored at room temperature, and crude DNA for the *bmp* gene of *T. pallidum*. The *bmp* gene was selected for analysis because it showed optimal GC content for primer design (13) and had highly specific and conserved sequences among *T. pallidum* subsp. *pallidum*, subsp. *endemicum*, and subsp. *pertenue*. Xiao et al. (13) also developed a LAMP assay for *bmp*, but it requires a 60-min reaction, excluding sample preparation time, due to the lack of loop primers, which accelerate the LAMP reaction. Becherer et al. (15, 18) developed a multiplex LAMP assay for the simultaneous detection of *T. pallidum* and *Haemophilus ducreyi* on a real-time PCR system. For point-of-care testing, simple methods and short detection times are preferable. In this study, we showed that the Dry-LAMP assay could detect *T. pallidum* DNA with a simple procedure, short detection time (within 60 min including sample preparation time), storage at room temperature, and equivalent sensitivity to the conventional LAMP assay. In addition, our LAMP assays showed no cross-reactivity to *Treponema denticola*, which is found in the oral microbiome (30) and can affect specificity when saliva specimens are used. Dry-LAMP assays can reduce the time, cost, and human resources needed to conduct analysis. In the clinical setting, especially in areas with limited resources, using Dry-LAMP assays is an option for a supportive diagnosis of syphilis. It is important to note that while the LAMP method showed a high total agreement rate with qPCR and nPCR when using saliva, it had a lower total agreement rate when using urine or blood. The LAMP method had lower sensitivity than qPCR and nPCR; thus, when using urine or blood samples with a low *T. pallidum* DNA copy number, the occurrence of false-negative results may be increased.

In this study, we also showed that saliva samples can be used for MLST of syphilis. Saliva has a higher positive rate of *T. pallidum* DNA than urine and blood and contains an adequate *T. pallidum* DNA load for MLST and MLSA. MLST can also be applied using non-invasive specimens such as oral and anal swabs (19, 20). Saliva can be self-collected in a more convenient and less uncomfortable manner than using swabs. Interestingly, TEN was also detected in saliva specimens as well as *T. pallidum* subsp. *pallidum*. Classically, TEN is endemic in hot and dry regions such as the Middle East and causes endemic syphilis (bejel). The clinical presentation of bejel is similar to syphilis (31); thus, it is difficult to distinguish bejel and syphilis based on clinical findings alone in non-endemic countries (32). Several reports have indicated that the local transmission of TEN occurs outside of traditional endemic countries such as Cuba (33, 34) and Japan (21, 27); however, the epidemic situation and transmission route of TEN in non-endemic areas remain unclear. Molecular surveillance using saliva could accelerate the accumulation of molecular epidemiology data of not only *T. pallidum* subsp. *pallidum* but also TEN.

The major limitation of this study is that there were missing serological data from multiple time points and our patient population did not cover the very early stage of syphilis. Thus, the diagnostic performance of molecular testing of non-invasive specimens for syphilis, especially sensitivity for primary and latent syphilis, could have been overestimated. In addition, the subjects of this study were patients in the outpatient setting and showed a high prevalence of syphilis. The utility of molecular testing of non-invasive specimens to screen asymptomatic individuals with an estimated low prevalence is not clear. Further study is necessary to determine the more accurate diagnostic performance and utility of molecular tests using non-invasive specimens to screen for syphilis.

### Conclusion

Although attention should be paid to the false-negative results, molecular analysis of non-invasive self-collected saliva samples could be used as a supplementary diagnostic test for syphilis, especially in patients who are reluctant to provide blood samples. The novel Dry-LAMP assay developed in this study is expected to be helpful in the clinical setting. Furthermore, saliva can be useful for the molecular surveillance of *T. pallidum* by MLST and MLSA.

## Data Availability

Sequence data for *tp0548* and *tp0856* are available in NCBI GenBank under accession numbers LC817247 and LC817248, respectively.

## SUPPLEMENTAL MATERIAL

Figure S1 jcm.00809-24-s0001.pdf Multilocus sequence analysis of *tp0548* and *tp0856*.

Table S1 jcm.00809-24-s0002.pdf Primers used in this study.

Table S2 jcm.00809-24-s0003.pdf Control DNA used in this study.

Table S3 jcm.00809-24-s0004.pdf Reference sequences used in this study for multilocus sequence analysis.

Table S4 jcm.00809-24-s0005.pdf Summary of the comparison of molecular testing using clinical specimens.

ASM does not own the copyrights to Supplemental Material that may be linked to, or accessed through, an article. The authors have granted ASM a non-exclusive, world-wide license to publish the Supplemental Material files. Please contact the corresponding author directly for reuse.
