# Supplementary material for: Prospective evaluation of non-invasive saliva specimens for the diagnosis of syphilis and molecular surveillance of Treponema pallidum
Source: J Clin Microbiol. 2024 Nov 6;62(12):e00809-24. doi: 10.1128/jcm.00809-24 (PMC11633093; doi:10.1128/jcm.00809-24)

Supplementary Figure 1. Multi-locus sequence analysis of *tp0548* and *tp0856*. A phylogenetic tree was constructed using the maximum likelihood method with bootstrap analyses (1,000 samples). The red arrow indicates the clinical sample in this study.

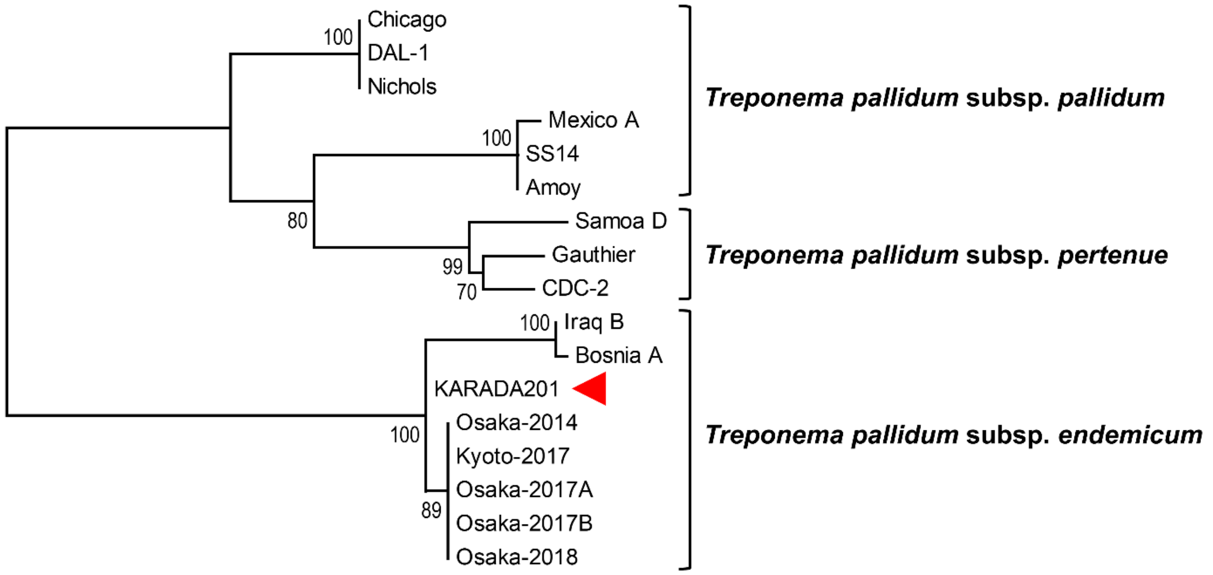

Supplement: Figure S1 — Multilocus sequence analysis of tp0548 and tp0856. [file jcm.00809-24-s0001.pdf]
