# Supplementary material for: Prospective evaluation of non-invasive saliva specimens for the diagnosis of syphilis and molecular surveillance of Treponema pallidum
Source: J Clin Microbiol. 2024 Nov 6;62(12):e00809-24. doi: 10.1128/jcm.00809-24 (PMC11633093; doi:10.1128/jcm.00809-24)
Supplement: Table S1 — Primers used in this study. [file jcm.00809-24-s0002.pdf]

Supplementary Table 1. Primers used in this study

Primer name	Target gene	Sequence (5′–3′)	Application	Reference
SY767F (*tp47* internal F)	*tp47*; *tp0574*	CAACACGGTCCGCTACGACTA	qPCR and nPCR	1
SY834R (*tp47* internal R)	*tp47*; *tp0574*	TGCCATAACTCGCCATCAGA	qPCR and nPCR	1
SY791T	*tp47*; *tp0574*	FAM–CGGTGATGACGCGAGCTACACCA–BHQ1	qPCR	1
qPCR *polA* F	*polA; tp0105*	GGTAGAAGGGAGGGCTAGTA	qPCR	2
qPCR *polA* R	*polA; tp0105*	CTAAGATCTCTATTTTCTATAGGTATGG	qPCR	2
qPCR *polA* probe	*polA; tp0105*	FAM-ACACAGCACTCGTCTTCAACTCC-BHQ1	qPCR	2
tp47 external F	*tp47*; *tp0574*	TTCTGCACGTAAGGTAAGC	nPCR	3
tp47 external R	*tp47; tp0574*	GTCAGCCTGTAGTATCCCG	nPCR	3
polA external F	*tp47*; *tp0574*	TGCGCGTGTGCGAATGGTGTGGTC	nPCR	4
polA external R	*polA; tp0105*	CACAGTGCTCAAAAACGCCTGCACG	nPCR	4
polA internal F	*polA; tp0105*	GGATTGCATCCGCACGATAC	nPCR	4
polA internal R	*polA; tp0105*	CAGCAGATGCAGATACCCCA	nPCR	4
tp0136 external F	*tp1036*	AACCCGTTAGCGCCCAACAT	nPCR	5
tp0136 external R	*tp1036*	TCCCAGCTCAGCCGAATCTC	nPCR	5
tp0136 internal F	*tp1036*	AGTGTCTTCCTCGTCCGTTC	nPCR	5
tp0136 internal R	*tp1036*	CACGTGGTGGTGTCAAACTT	nPCR	5
tp0548 external F	*tp0548*	TGGGGCACTAAACCGGAAGA	nPCR	5
tp0548 external R	*tp0548*	TACGGGCATTTGCGGATAGG	nPCR	5
tp0548 internal F	*tp0548*	GCGGTCCCTATGATATCGTGT	nPCR	5
tp0548 internal R	*tp0548*	GAGCCACTTCAGCCCTACTG	nPCR	5
tp0705 external F	*tp0705*	GGTCTATATGCAGCCCTTCTTC	nPCR	5
tp0705 external R	*tp0705*	GCTTGAGAACGATACCGGATAC	nPCR	5
tp0705 internal F	*tp0705*	TGCGGCTTATCCTGATGAATAG	nPCR	5
tp0705 internal R	*tp0705*	TATTCTGCGGCGTTGGATAG	nPCR	5
bmp 6 F3	*bmp*	CCGTTTCATTCACGTCCGAA	LAMP	This study
bmp 6 B3	*bmp*	GCGAAAGCGCAAGAGTTTG	LAMP	This study
bmp 6 FIP	*bmp*	TACTGCCACGCAGCTTGGTCCACCAACTCCCATGCCAA	LAMP	This study
bmp 6 BIP	*bmp*	CAGAGTAACCCGCCTGTGCATTCCGGTGTCAAAGCCAC	LAMP	This study
bmp 6 LF	*bmp*	TGACGATGGAGGCGTTGCT	LAMP	This study
bmp 6 LB	*bmp*	CCATTTGCCTTACTCTTAATGTCAGCC	LAMP	This study

LAMP, loop-mediated isothermal amplification; nPCR, nested PCR; qPCR, quantitative PCR

1. Gayet-Ageron A, et al. Assessment of a real-time PCR test to diagnose syphilis from diverse biological samples. Sex Transm Infect. 2009;85(4):264-9.

2. Heymans R, et al. Clinical Value of Treponema pallidum Real-Time PCR for Diagnosis of Syphilis. J Clin Microbiol. 2010; 48(2): 497–502.

3. Wang C, et al. Sensitive detection of Treponema pallidum DNA from the whole blood of patients with syphilis by

the nested PCR assay. Emerg Microbes Infect. 2018;7(1):83.
4. Liu H, et al. New Tests for Syphilis: Rational Design of a PCR Method for Detection of Treponema pallidum in Clinical Specimens Using Unique Regions of the DNA Polymerase I Gene J Clin Microbiol. 2001;39(5): 1941–1946.
5. Grillová L, et al. Molecular characterization of Treponema pallidum subsp. pallidum in Switzerland and France with a new multilocus sequence typing scheme. PLoS One. 2018;13(7):e0200773.
