# Supplementary material for: Prospective evaluation of non-invasive saliva specimens for the diagnosis of syphilis and molecular surveillance of Treponema pallidum
Source: J Clin Microbiol. 2024 Nov 6;62(12):e00809-24. doi: 10.1128/jcm.00809-24 (PMC11633093; doi:10.1128/jcm.00809-24)
Supplement: Table S2 — Control DNA used in this study. [file jcm.00809-24-s0003.pdf]

Supplementary Table 2. Control DNA used in this study

No.	Species	Resource
1	*Acinetobacter baumanii*	National BioResource Project
2	*Aggregatibacter segnis*	National BioResource Project
3	*Aspergillus flavaus*	National BioResource Project
4	*Aspergillus fumigatus*	National BioResource Project
5	*Aspergillus niger*	National BioResource Project
6	*Basidiobolus meristosporus*	National BioResource Project
7	*Burkholderia pseudomallei*	National BioResource Project
8	*Candida albicans*	National BioResource Project
9	*Candida glabrata*	National BioResource Project
10	*Candida krusei*	National BioResource Project
11	*Candida parapsilosis*	National BioResource Project
12	*Candida tropicalis*	National BioResource Project
13	*Chlamydia trachomatis*	Vircell S.L.
14	*Citrobacter frendii*	National BioResource Project
15	*Cladosporium carrionii*	National BioResource Project
16	*Corynebacterium diphtheriae*	National BioResource Project
17	*Cryptococcus gatii*	National BioResource Project
18	*Cryptococcus neoformans*	National BioResource Project
19	*Enterobacter cloacae*	National BioResource Project
20	*Epidermophyton floccosum*	National BioResource Project
21	*Escherichia coli*	National BioResource Project
22	*Fonsecaea pedrosoi*	National BioResource Project
23	*Haemophilus haemolyticus*	National BioResource Project
24	*Haemophilus influenzae*	National BioResource Project
25	*Haemophilus parainfluenzae*	National BioResource Project
26	*Herpes simplex virus type 1*	Vircell S.L.
27	*Herpes simplex virus type 2*	Vircell S.L.
28	*Klebsiella pneumoniae*	National BioResource Project
29	*Legionella pneumoniae*	National BioResource Project
30	*Madurella mycetomi*	National BioResource Project
31	*Malassezia furfur*	National BioResource Project
32	*Microsporum ferrugineum*	National BioResource Project
33	*Moraxella catarrhalis*	National BioResource Project
34	*Mucor circinelloides*	National BioResource Project
35	*Mycoplasma genitalium*	Vircell S.L.
36	*Mycoplasma hominis*	Vircell S.L.

37	*Mycoplasma pneumoniae*	National BioResource Project
38	*Neisseria gonorrhoeae*	Vircell S.L.
39	*Nocardia farcinica*	National BioResource Project
40	*Proteus mirabilis*	National BioResource Project
41	*Pseudomonas aeruginosa*	National BioResource Project
42	*Rhizopus oryzae*	National BioResource Project
43	*Serratia marcescens*	National BioResource Project
44	*Spirotrichum purpureum*	National BioResource Project
45	*Staphylcoccus aureus*	National BioResource Project
46	*Staphylcoccus aureus*	National BioResource Project
47	*Staphylcoccus epidermidis*	National BioResource Project
48	*Stenotrophomonas maltophilia*	National BioResource Project
49	*Strepotococcus mutans*	National BioResource Project
50	*Strepotococcus pneumoniae*	National BioResource Project
51	*Streptococcus agalactiae*	National BioResource Project
52	*Streptococcus pyogenes*	National BioResource Project
53	*Treponema denticola*	National BioResource Project
54	*Treponema pallidum*	Vircell S.L.
55	*Trichophyton rubrum*	National BioResource Project
56	*Ureaplasma parvum*	Vircell S.L.
57	*Ureaplasma urealyticum*	Vircell S.L.
