# Supplementary material for: Prospective evaluation of non-invasive saliva specimens for the diagnosis of syphilis and molecular surveillance of Treponema pallidum
Source: J Clin Microbiol. 2024 Nov 6;62(12):e00809-24. doi: 10.1128/jcm.00809-24 (PMC11633093; doi:10.1128/jcm.00809-24)
Supplement: Table S3 — Reference sequences used in this study for multilocus sequence analysis. [file jcm.00809-24-s0004.pdf]

Supplementary Table 3. Reference sequences used in this study for multi-locus sequence analysis

No.	Species	Strain	Gene	NCBI accession no.	Gene	NCBI accession no.
1	*Treponema pallidum* subsp. *endemicum*	KARADA201	*tp0856*	LC817248	*tp0548*	LC817247
2	*Treponema pallidum* subsp. *endemicum*	Iraq B	*tp0856*	CP032303	*tp0548*	CP032303
3	*Treponema pallidum* subsp. *endemicum*	Bosnia A	*tp0856*	CP007548	*tp0548*	CP007548
4	*Treponema pallidum* subsp. *endemicum*	Osaka-2014	*tp0856*	LC383799	*tp0548*	LC430604
5	*Treponema pallidum* subsp. *endemicum*	Kyoto-2017	*tp0856*	LC430601	*tp0548*	LC430606
6	*Treponema pallidum* subsp. *endemicum*	Osaka-2017A	*tp0856*	LC383801	*tp0548*	LC430605
7	*Treponema pallidum* subsp. *endemicum*	Osaka-2017B	*tp0856*	LC430602	*tp0548*	LC430607
8	*Treponema pallidum* subsp. *endemicum*	Osaka-2018	*tp0856*	LC430603	*tp0548*	LC430608
9	*Treponema pallidum* subsp. *pallidum*	Nichols	*tp0856*	CP004010	*tp0548*	CP004010
10	*Treponema pallidum* subsp. *pallidum*	DAL-1	*tp0856*	CP003115	*tp0548*	CP003115
11	*Treponema pallidum* subsp. *pallidum*	Chicago	*tp0856*	CP001752	*tp0548*	CP001752
12	*Treponema pallidum* subsp. *pertenue*	Samoa D	*tp0856*	CP002374	*tp0548*	CP002374
13	*Treponema pallidum* subsp. *pertenue*	CDC-2	*tp0856*	CP002375	*tp0548*	CP002375
14	*Treponema pallidum* subsp. *pertenue*	Gauthier	*tp0856*	CP002376	*tp0548*	CP002376
15	*Treponema pallidum* subsp. *pallidum*	Amoy	*tp0856*	CP015162	*tp0548*	CP015162
16	*Treponema pallidum* subsp. *pallidum*	SS14	*tp0856*	CP111135	*tp0548*	CP111135
17	*Treponema pallidum* subsp. *pallidum*	Mexico A	*tp0856*	CP003064	*tp0548*	CP003064
