# Supplementary material for: Prospective evaluation of non-invasive saliva specimens for the diagnosis of syphilis and molecular surveillance of Treponema pallidum
Source: J Clin Microbiol. 2024 Nov 6;62(12):e00809-24. doi: 10.1128/jcm.00809-24 (PMC11633093; doi:10.1128/jcm.00809-24)
Supplement: Table S4 — Summary of the comparison of molecular testing using clinical specimens. [file jcm.00809-24-s0005.pdf]

Supplementary Table 4. Summary of the comparison of molecular testing using clinical specimens

Saliva (n = 163)		Dry-LAMP		Total agreement rate (%)	κ coefficient
		Positive	Negative		
**Saliva (n = 163)**					
Quantitative PCR	Positive	46	7	95.7	0.90
	Negative	0	110		
Nested PCR	Positive	46	6	96.3	0.91
	Negative	0	112		
Conventional LAMP	Positive	46	3	98.2	0.96
	Negative	0	114		
**Urine (n = 152)**					
Quantitative PCR	Positive	6	21	86.2	0.32
	Negative	0	125		
Nested PCR	Positive	5	18	87.5	0.30
	Negative	1	128		
Conventional LAMP	Positive	6	7	95.4	0.61
	Negative	0	139		
**Blood (n = 137)**					
Quantitative PCR	Positive	1	30	78.1	0.05
	Negative	0	106		
Nested PCR	Positive	1	33	75.9	0.04
	Negative	0	103		
Conventional LAMP	Positive	1	3	97.8	0.39
	Negative	0	133		

CI, confidence interval; LAMP, loop-mediated isothermal amplification
